# Cerium Oxide Nanoparticle Administration to Skeletal Muscle Cells under Different Gravity and Radiation Conditions

**DOI:** 10.1021/acsami.1c14176

**Published:** 2021-08-19

**Authors:** Giada Graziana Genchi, Andrea Degl’Innocenti, Chiara Martinelli, Matteo Battaglini, Daniele De Pasquale, Mirko Prato, Sergio Marras, Giammarino Pugliese, Filippo Drago, Alessandro Mariani, Michele Balsamo, Valfredo Zolesi, Gianni Ciofani

**Affiliations:** † Istituto Italiano di Tecnologia, Smart Bio-Interfaces, Viale Rinaldo Piaggio 34, 56025 Pontedera (Pisa), Italy; ‡ Scuola Superiore Sant’Anna, The BioRobotics Institute, Viale Rinaldo Piaggio 34, 56025 Pontedera (Pisa), Italy; § Istituto Italiano di Tecnologia, Materials Characterization, Via Morego 30, 16163 Genova, Italy; ∥ Istituto Italiano di Tecnologia, Nanochemistry, Via Morego 30, 16163 Genova, Italy; ⊥ Kayser Italia S.r.l., Via di Popogna 501, 57128 Livorno, Italy

**Keywords:** cerium oxide nanoparticles, skeletal muscle cells, microgravity, radiations, transcriptome, gene ontology, *uncoupling protein 2 (Ucp2)*

## Abstract

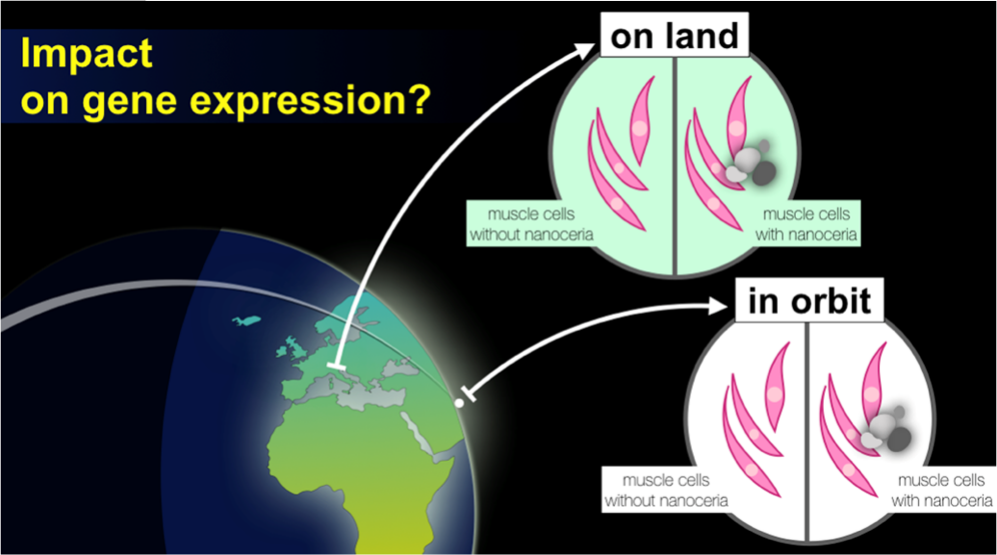

For their remarkable biomimetic properties implying strong modulation of the intracellular and extracellular redox state, cerium oxide nanoparticles (also termed “nanoceria”) were hypothesized to exert a protective role against oxidative stress associated with the harsh environmental conditions of spaceflight, characterized by microgravity and highly energetic radiations. Nanoparticles were supplied to proliferating C2C12 mouse skeletal muscle cells under different gravity and radiation levels. Biological responses were thus investigated at a transcriptional level by RNA next-generation sequencing. Lists of differentially expressed genes (DEGs) were generated and intersected by taking into consideration relevant comparisons, which led to the observation of prevailing effects of the space environment over those induced by nanoceria. In space, upregulation of transcription was slightly preponderant over downregulation, implying involvement of intracellular compartments, with the majority of DEGs consistently over- or under-expressed whenever present. Cosmic radiations regulated a higher number of DEGs than microgravity and seemed to promote increased cellular catabolism. By taking into consideration space physical stressors alone, microgravity and cosmic radiations appeared to have opposite effects at transcriptional levels despite partial sharing of molecular pathways. Interestingly, gene ontology denoted some enrichment in terms related to vision, when only effects of radiations were assessed. The transcriptional regulation of mitochondrial uncoupling protein 2 in space-relevant samples suggests perturbation of the intracellular redox homeostasis, and leaves open opportunities for antioxidant treatment for oxidative stress reduction in harsh environments.

## Introduction

Skeletal muscle tissue alterations such as atrophy, muscle force decrease, and shift in muscle fiber composition occur during aging, pathology onset, radiation exposure, and mechanical unloading.^1−5^ A fast deterioration of skeletal muscle tissue, in particular, affects astronauts who are exposed both to gravitational unloading (hereafter termed microgravity or “μ*g*”) and to cosmic radiation during spaceflight. For this reason, a variety of strategies for muscular maintenance *in vitro* and *in vivo* has also been devised for terrestrial benefit, including physical exercise,^6^ mechanical stimulation in the form of vibrations^7^ and pressure application,^1^ electrical stimulation,^8^ exposure to hypergravity,^9−11^ administration of soluble factors (such as activin type IIB receptor,^12^ recombinant myokine irisin,^13^ and myostatin antibody YN41^14^), and even genetic transduction finalized to the overexpression of nucleic acids (such as a long noncoding RNA termed lncMUMA).^15^ A common approach against skeletal muscle waste due to mechanical unloading also consists of the supply of antioxidant compounds, like for instance (-)-epicatechin,^16^ lecithin,^17^*N*-acetylcysteine,^18^ complex mixtures of polyphenols associated with other antioxidants (such as vitamin E, selenium, and omega-3 fatty acids),^19^ or even seed extracts (from *Oenothera odorata*).^20^ Besides targeting autophagic flux^21^ and myostatin signaling,^2,10,12,14^ the most recent research indeed focuses on the role of oxidative stress (OS) due to excess reactive oxygen species (ROS) and mitochondrial dysregulation in skeletal muscle degeneration under real or simulated microgravity (s-μ*g*).^12,18,22,23^ As human permanence in the low Earth orbit undergoes increasing duration and opens to interplanetary travel, the understanding of the biological effects of mechanical unloading necessitates deepening by careful consideration of the consequences of exposure to highly energetic cosmic radiations that to date is largely obscure and apparently follows divergent molecular pathways in comparison to microgravity.^24−28^

Based on the large body of evidence of the antioxidant and radioprotective properties of cerium oxide nanoparticles (also termed “nanoceria” (NC)) accumulated on ground,^29−35^ and assimilating long-standing catalytic activity of this typology of inorganic materials to that one of superoxide dismutase and catalase,^36−38^ we decided to test them for skeletal muscle tissue protection in space. In a previous study, our group explored NC effects on differentiating muscle cell cultures held on board the International Space Station (ISS).^39^ The collected transcriptional evidence demonstrated cellular adaptation to nanoparticle administration and gravitational unloading, and suggested regulation of biological processes related to aging, fat tissue development, and mesodermal tissue proliferation.^39^ In another pilot study from our group, NC was also shown to decrease cell death and DNA fragmentation while promoting stemness and tissue regeneration in planarian worms exposed to low-dose radiations,^40^ thus demonstrating potential radioprotective activity relevant to spaceflight.

This work aimed at verifying putative protection of NC against deleterious space environment effects on proliferating C2C12 mouse skeletal muscle cells, which were cultured on board the ISS in two different experimental configurations, meaning either exposure to microgravity and cosmic radiations or to artificially obtained Earth gravity (1*g* by centrifugation) and cosmic radiations. Samples collected in space were compared to samples obtained later on ground by the application of the same time and temperature profiles of spaceflight. On-ground analyses by RNA next-generation sequencing (RNA-seq) enabled the identification of differentially expressed genes (DEGs) among paired experimental classes. Sets of DEGs underwent hierarchical clustering, comparison through Venn diagrams, and representation by gene ontology (GO) graphs. The results denoted important transcriptional regulation of mitochondrial and nuclear compartments; they demonstrated opposite effects of gravitational unloading and cosmic radiations and evidenced the need for further optimization of NC delivery modes in view of exploitation of their remarkable antioxidant properties in the space environment.

## Experimental Methods

### Preparation of Nanoparticle Dispersions

Cerium oxide nanoparticles (NC, Sigma 544841) were dispersed at a concentration of 10 mg/mL in ultrapure water. The dispersion was sonicated for 1 min at 8 W with a tip sonicator (Bandelin). Then, 5 mg/mL NC dispersions in 50% fetal bovine serum (FBS, Sigma F4135) were prepared by incubation for 1 h under mild shaking to promote nanoparticle coating with serum proteins. At the end of this process, sonication was performed for 5 min with a Branson sonication bath to obtain highly homogeneous dispersions. For material characterization, dispersions were prepared in ultrapure water, in 10% FBS solution in ultrapure water, and in a complete cell culture medium (composition of the latter is described in the “Cell culture in-flight hardware” paragraph).

### Material and Dispersion Characterization

Transmission electron microscopy (TEM) imaging was conducted on uncoated and FBS-coated nanoparticles by dispersion in ultrapure water at a final concentration of 1 mg/mL NC and by dropping 50 μL of dispersions onto carbon-coated copper grids. Imaging was performed in a bright-field modality with a Tecnai G2 F20 TWIN TMP transmission electron microscope set at 200 kV.

X-ray diffraction (XRD) analysis was performed on uncoated nanoparticles with a Rigaku SmartLab X-ray powder diffractometer equipped with a 9 kW Cu Kα rotating anode operating at 40 kV and 150 mA, a D\teX Ultra 1D silicon strip detector, and five-axis goniometer. The diffraction pattern was collected in Bragg–Brentano geometry over an angular range 2θ = 20–100°, with a step size of 0.02°. The measurement was carried out at room temperature using a zero-diffraction silicon substrate. Size-strain analysis was carried out using the whole-powder-pattern decomposition (WPPD) technique based on the Pawley algorithm. Fundamental parameter (FP) profile fitting was used to simulate the instrument contribution. All parameters were refined by the least-squares method, using PDXL 2.8.1.1 software from Rigaku.

X-ray photoelectron spectroscopy (XPS) analysis was performed on uncoated nanoparticles deposited onto an indium pellet with a Kratos Axis Ultra DLD spectrometer, equipped with a monochromatic Al Kα source operating at 15 kV and 20 mA. A wide scan spectrum was acquired with 160 eV pass energy, while a high-resolution narrow scan spectrum was obtained with constant 10 eV pass energy and steps of 0.1 eV. Photoelectrons were detected at a take-off angle φ = 0° with respect to the surface normal. The charging shift was calibrated with the binding energy of the C 1s as a baseline (284.8 eV). Data were acquired at a pressure lower than 7·10^–9^ Torr in the analysis chamber, and then they were converted to VAMAS format and processed using CasaXPS software, version 2.3.22.

Thermogravimetric analysis (TGA) was conducted with a TA instruments Q500 thermal analyzer under a 50 mL/min nitrogen flux on ∼5 mg of nanoparticle powders, obtained by lyophilization of 1 mL of both uncoated and FBS-coated nanoparticle dispersions in ultrapure water with a Labconco Freezone 2.5 Plus freeze dryer. The samples were analyzed after a 5 min equilibration at +30 °C, and then the temperature was increased by a +5 °C/min ramp-up to +1000 °C.

Dynamic light scattering (DLS) measurements were performed with a Malvern Instruments Zeta-sizer NanoZS90 on 100 μg/mL NC dispersions in ultrapure water, in 10% FBS in ultrapure water, and in complete cell culture medium after exposure to both normal gravity and simulated microgravity (described in the following paragraph). Measurements were conducted every two days over a 14-day period at +37 °C. ζ-potential measurements were carried out in NC dispersions in ultrapure water (pH 5.5), and the conductivity was adjusted in the range of 30–100 μS/cm. Hydrodynamic diameter (HD) and ζ-potential values are represented as average ± standard deviation of three different measurements, each with 15 readings.

### Simulated Microgravity

Simulated microgravity conditions (hereafter also termed “s-μ*g*”) were applied by operating a random positioning machine (RPM, Airbus 2.0) at 20°/s for studies on nanoparticle dispersion stability, and at 8–20°/s (speed change: every 5 s) for nanoparticle internalization studies with C2C12 mouse myoblasts. To this purpose, containers completely filled with liquid media were used by positioning within 22 mm from the RPM center of rotation: microcentrifuge tubes for stability studies and *ad hoc*-prepared polydimethylsiloxane (silicone) vessels sealed with transparent adhesive films (Bio-Rad MSB1001) for internalization studies. The RPM home frame position was the one having the sample holder aligned with the Earth gravity vector (with both frames set perpendicular to the Earth gravity vector).

### Cell Culture for Nanoparticle Internalization Studies

To perform nanoparticle internalization studies by immunostaining and confocal microscopy imaging, C2C12 myoblasts (ATCC CRL-1772) at passage 6–10 were seeded at a density of 10,000 cells/cm^2^ on Thermanox substrates (1.05 × 2.20 cm^2^) positioned at the bottom of silicone multiwell plates, and incubated for 12 h at +37 °C prior to treatment. The latter consisted of the administration of the cell culture medium (1.3 mL) either added or not with NC at a concentration of 100 μg/mL (followed by vessel sealing with adhesive films) and exposure to either 1 *g* or to s-μ*g*. Cell cultures were imaged after 6 and 24 h of treatment by application of the protocol reported in the paragraph “Immunostaining and confocal microscopy”. To perform nanoparticle internalization studies by inductively coupled plasma-optical emission spectroscopy (ICP-OES) as described later, cell cultures were seeded on tissue culture-treated polystyrene disks (22 mm radius, previously obtained by laser cutting of commercial Petri dishes and positioned in silicone vessels) for 12 h at +37 °C prior to treatment. The latter again consisted in the administration of the cell culture medium (8.6 mL, in this case), either added or not with NC, and exposure to either 1 *g* or to simulated μ*g*. ICP-OES analysis was conducted on cultures after 48 h of treatment, corresponding to the total duration of cell culture exposure to NC-added cell culture medium in space, before medium refresh without NC was performed.

After exposure to normal gravity and to simulated microgravity, cell cultures, either treated or not with nanoceria (NC), were fixed with 4% formaldehyde solution (Sigma 252549) in phosphate-buffered saline (PBS) with Ca^2+^/Mg^2+^ (Gibco 14040141) for 20 min at +4 °C. Then, the cells were permeabilized with a 0.1% Triton X-100 (Sigma T8787) solution in PBS for 20 min. Saturation of aspecific antigenic sites was conducted with a 10% goat serum (GS, Gibco 16210072) solution in PBS for 30 min, then an incubation with a 1:200 v/v dilution of primary antibodies (rabbit anti-caveolin, Abcam 2910, or mouse anti-clathrin, Abcam 2731) in 10% GS followed for 2 h at +37 °C. The samples were then rinsed three times with 10% GS (5 min each rinse). They were also incubated with a solution containing a 1:500 v/v dilution of secondary antibodies (TRITC-goat anti-rabbit, Invitrogen 2769 or TRITC-goat anti-mouse, Life Technologies A16071), 1:100 v/v dilution of Oregon Green 488-phalloidin (Life Technologies 07466), and 1:100 v/v Hoechst dye (Life Technologies H21486) for 1 h at room temperature. Finally, the samples were rinsed twice with PBS and imaged with a Nikon Ti-E confocal microscope. Five images of optical fields were collected for each sample, and semiautomated image analysis for the measurement of Pearson’s correlation coefficient on the fluorescence signal from proteins and NC was conducted with ImageJ software to elucidate possible nanoparticle internalization modalities.

To perform inductively coupled plasma-optical emission spectroscopy (ICP-OES), cell pellets were obtained by trypsinization and two sequential centrifugation runs at 700*g* were performed for 10 min by pellet resuspension in PBS to ensure the removal of free, non-internalized NC prior to the second centrifugation run. ICP-OES was conducted with a Thermo Fisher Scientific iCAP 7600 DUO Thermo spectrometer upon dissolution of the cell culture pellets in HCl/HNO_3_ (3:1 v/v ratio). The spectrometer was operated under Ar flow with the following parameters: 1150 W RF power, 0.5 L/min nebulizer gas flow, 12 L/min coolant gas flow, and 0.5 L/min auxiliary gas flow. Measurements were conducted in an axial mode with a 13 s exposure time for readings at a wavelength of 404.076 and 535.353 nm. Data are presented as an average of the readings at both wavelengths.

### Cell Culture in-Flight Hardware

**Figure 1:**
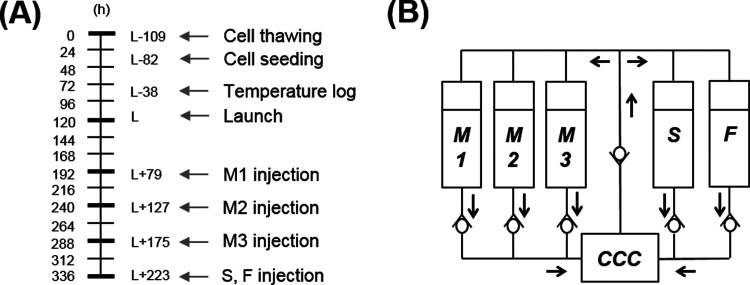
In-flight experiment. (A) Experiment timeline with the indication of relevant time points before and after launch. (B) Diagram of the flow circuit of experiment units, where “CCC” stands for the cell culture chamber, “M” for the cell culture medium, “S” for saline, and “F” for the fixative solution. M1: ±NC for cells that were either treated (+ NC) or not (−NC) with nanoceria, M2: −NC, and M3: −NC.

C2C12 myoblasts at passage 6–10 were seeded at a density of 1,500 cells/cm^2^ on Thermanox coverslips in silicone multiwell plates at L-90 h, where “L” stands for launch time. A synthetic description of the experiment timeline is reported in Figure 1A. At L-42 h, the samples were transferred to 12 experiment units (EUs, KEU-ST developed by Kayser Italia (KI) and qualified for flight to the ISS) holding five reservoirs with 1.3 mL capacity, which were filled as follows: reservoirs M1–3 with cell culture medium (CO_2_-independent medium, Gibco 18045088, added with 10% FBS, 100 U/mL penicillin-100 μg/mL streptomycin, Gibco 15140122), and either added or not with FBS-coated nanoparticles at a final concentration of 100 μg/mL; reservoir S with saline solution (PBS with Ca^2+^/Mg^2+^, Gibco 14040141) for cell rinse prior to fixation, and reservoir F with a fixative solution (RNAlater, Ambion AM7020) for nucleic acid preservation. EU flow diagram is reported in Figure 1B. EUs (*n* = 3 for each experimental class) were enclosed in experiment containers (ECs, KIC-SL developed by KI and qualified for flight to the ISS), ensuring second-level containment and interface with the European Space Agency (ESA) Kubik incubator facility on board the ISS. Passive conditioned temperature (+25 to +30 °C) was kept from handover until hardware installation in the Kubik incubator (set at +37 °C) on board the ISS. The temperature was recorded with iButton data loggers every 10 min from completion of hardware assembly and verification (L-38 h, *t* = 44 h from seeding) until the experiment end. Upload occurred with a Dragon/Falcon 9 vector (SpaceX CRS-17 mission). Once ECs were placed in the Kubik incubator set at +37 °C, five fluidic activations were automatically performed. Activation 1 was performed at L + 79 h (after EU powering in the Kubik incubator), and it provided a fresh culture medium either with or without NC to the cultures. Activations 2 and 3 were, respectively, performed at L + 127 and L + 175 h without NC supply, which implied that the cultures were exposed to NC for only 48 h under microgravity conditions. Activations 4 and 5 were in particular performed at L + 223 h (activation 5 with 5 min delay). After 2 h from fixation, the ECs were transferred to the Minus Eighty-Degree Laboratory Freezer for ISS (MELFI) and stowed at −80 °C until re-entry. Once the space experiment flight time and temperature profiles were known, the whole experiment was repeated on ground in the KEU-ST and KIC-SL hardware with the support of a Kubik Interface Simulation Station (KISS) module placed inside a laboratory tissue culture incubator.

### Transcriptomics

A total of six experimental classes result from studying NC and space/space radiations, namely: A (−NC, μ*g* in space), B (+NC, μ*g* in space), C (−NC, 1 *g* in space), D (+NC, 1 *g* in space), E (-NC, 1 *g* on ground), and F (+ NC, 1 *g* on ground). From the 15 possible pairwise comparisons, the following nine comparisons were selected (control class reported as the first term of each comparison): (E *vs* F) studying the effects of NC *per se*; (A *vs* B) studying the effects of NC against space; (C *vs* D) studying the effects of NC against space radiations; (E *vs* A) studying the effects of space *per se*; (F *vs* B) studying the effects of space on NC-treated cells; (E *vs* C) studying the effects of space radiations *per se*; (F *vs* D) studying the effects of space radiations on NC-treated cells; (C *vs* A) studying the effects of microgravity *per se* (with space radiations as a background); (D *vs* B) studying the effects of microgravity on NC-treated cells (with space radiations as a background).

After hardware deintegration, cell cultures were imaged by optical microscopy in phase-contrast mode with a Nikon Eclipse Ti microscope, and temperature profile was recovered from data loggers for verification and for experiment repetition on ground within six EUs (three treated with NC and three untreated). The cell cultures were transferred to 2 mL test tubes, covered with their supernatant (containing over 70% of RNAlater), and centrifuged at 16 000*g* for 15 min at +4 °C. The supernatant was carefully removed, leaving a volume of 100 μL to prevent cell loss. A MirVana PARIS kit (Ambion AM1556) was used for cell lysis, as well as for following RNA extraction and purification procedures. Cell lysis was performed by pipetting ice-cold cell disruption buffer (300 μL) on the cell culture-bearing support by vortexing for 30 s and by placing samples on the ice every 10 s. A 1.5% β-mercaptoethanol denaturing solution (400 μL) was added to each tube; after a 5 min incubation on ice, an acid-phenol:chloroform solution (800 μL) was added to the lysate. After a 30 s agitation with vortex, the support was removed and samples were centrifuged at 10 000*g* for 20 min. The aqueous phase (700 μL) from each sample was transferred to another clean tube, and 1.25 volumes of 99.9% ethanol (875 μL) were added. Each solution was then loaded twice on a filter column in a collection tube and centrifuged at 15 000*g* for 70 s. The eluate was discarded, and complete miRNA Wash Solution 1 (700 μL) was added for centrifugation at 15 000*g* for 1 min. The eluate was again discarded, and complete Wash Solution 2/3 (500 μL) was added twice for centrifugation at 15 000*g* for 1 min. The eluate was again discarded, and the filter column was fully dried prior to transfer to a clean collection tube. RNAse-free water (40 μL) was deposited in each filter column and incubated for 10 min, and then total RNA (including small RNAs) was eluted by centrifugation at 15 000*g* for 5 min.

Upon total RNA isolation, spectrophotometric measurements were performed with a NanoDrop 2000 spectrophotometer (Thermo Scientific) to assess sample quality and yield before downstream transcriptomic analyses through RNA-seq. RNA concentrations resulted to be about 10 ng/μL (as reported in Table S1). RNA preparations were processed according to the Ultra-Low Input RNA-Seq (GeneWiz) pipeline, cf. https://web.genewiz.com/ultra-low-input-case-study. Standard libraries were prepared with the NEBNext Ultra RNA Library Prep Kit for Illumina (New England Biolabs). Briefly, polyadenylated fractions were enriched, fragmented, and reverse-transcribed (first- and second-strand cDNAs). Ends were repaired, 5′-phosphorylated, and poly(A)-tailed. The resulting sequences were ligated to universal adapters and polymerase chain reaction (PCR)-amplified. Small RNA (chiefly miRNA) libraries were prepared aside by ligating adapters selectively to those 5′- and 3′-ends processed by the endoribonuclease Dicer. The resulting sequences were also reverse-transcribed and amplified by PCR. Yields were determined through the Qubit DNA assay (Thermo Fisher Scientific) and NanoDrop, whereas size distribution was assessed with a 2100 Bioanalyzer (Agilent). Viable sequencing templates were quantified *via* real-time PCR. Sequencing was done on a HiSeq. 2500 (Illumina) sequencer, in 2 × 250 bp paired-end configuration. Raw sequence data generated from the sequencer were converted to FASTQ files. After initial quality controls, low-quality data were removed, and reads were trimmed to remove possible adapter sequences using Trimmomatic v.0.36.^41^ For small RNA samples, only trimmed reads with a length comprised between 15 and 31 nucleotides were retained.

### Bioinformatics

Polished sequences from standard libraries were mapped to the reference mouse genome (GRCm38.p6) found at Ensembl^42^ with STAR aligner^43^ v.2.5.2b, and BAM files were thus generated. Unique gene hit counts for genes were computed *via* featureCounts^44^ from the Subread package^45^ v.1.5.2. Only unique reads falling within exon regions were counted. Trimmed sequences from small RNA samples were instead searched against those of known miRNAs, available at miRBase 21,^46^ and the ones finding no relevant match within the database were considered to be potentially novel small RNAs. Differential gene expression analysis was conducted with DESeq. 2.^47^ For each of the selected comparisons between couples of experimental classes, only those transcripts that displayed an expression log_2_ fold change (FC) > abs(1) and that were supported by a Wald test-generated adjusted *p*-value < 0.05 (or by an empirical analysis of the differential gene expression test *p*-value < 0.05 for small RNAs) were deemed to be differentially expressed (specifically: downregulated if their FC < −2, and upregulated if their FC > 2). Nine lists of DEGs were obtained, along with heatmaps and scatter (volcano) plots. While small RNA DEGs were not further analyzed, standard differentially expressed gene (DEG) lists were intersected in multiple ways *via* scripting. For each subset of the resulting Venn diagrams, coherence was also evaluated. A DEG was considered coherent if systematically upregulated (or systematically downregulated) in all parent sets of the subset in which the DEG was found. In each Venn diagram, coherence is only defined for genes located at the intersection between two or more parent sets. To understand the biological effects of our experimental variables on the transcriptome, gene ontology (GO) investigations were performed for all subsets of our Venn diagrams using GOrilla:^48^ two unranked lists of genes were provided to the algorithm, using all mouse genes (retrieved at Ensembl BioMart^49^) as a background list. REVIGO^50^ was used to visualize selected GO results. In particular, GO terms for biological processes (defining high-level biological dynamics), functions (describing precise molecular events), and components (pointing out particular biological compartments, organelles, or structures) were characterized. Thresholds for statistical significance on GOrilla results were 0.05 for both (GOrilla-generated) *p*- and *q*-values.

## Results and Discussion

### Nanoparticle Characterization

**Figure 2:**
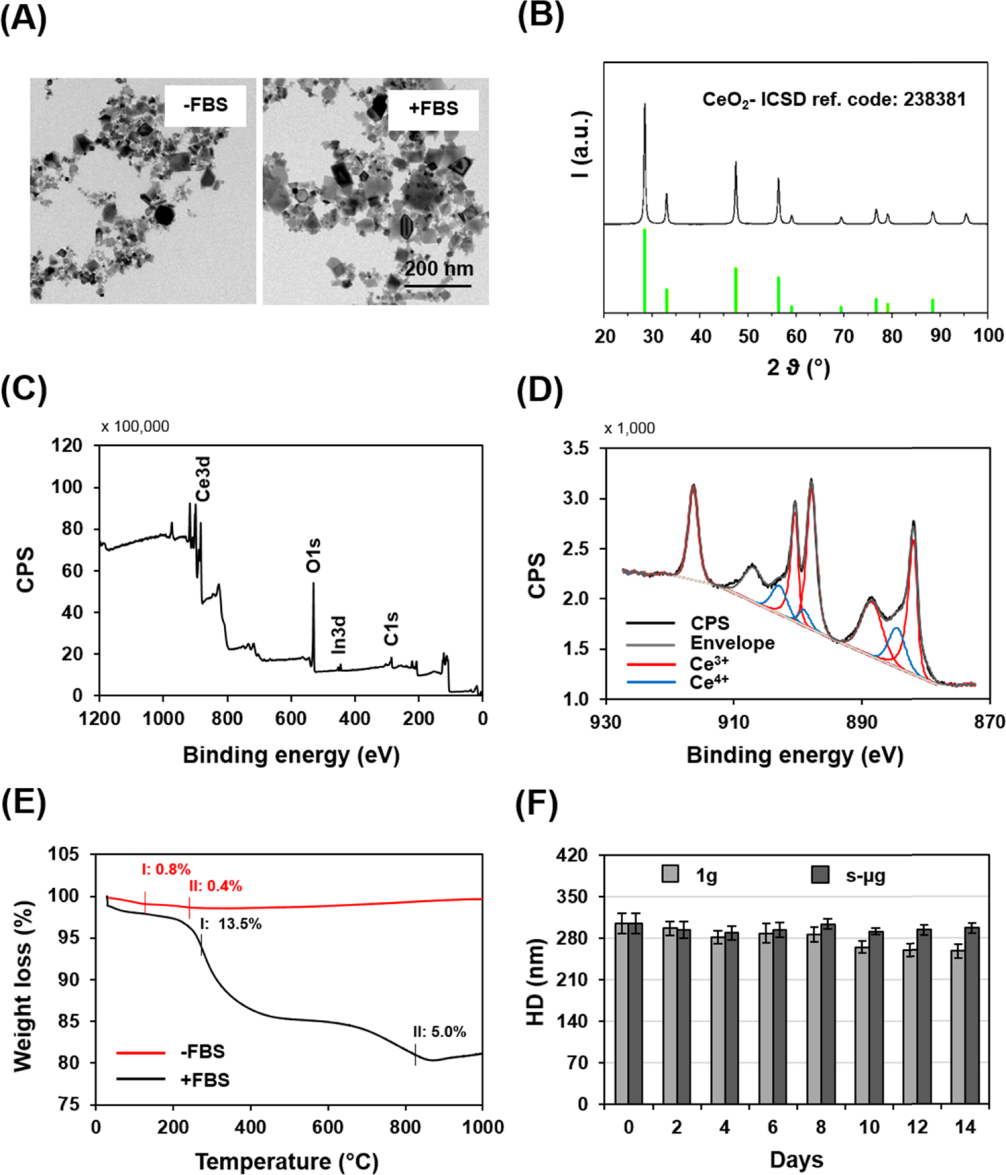
Characterization of cerium oxide nanoparticles (NC) and of their dispersions. (A) Electron microscopy image of nanoparticles before and after coating with fetal bovine serum (FBS, respectively indicated as −FBS and +FBS). (B) X-ray diffraction pattern of uncoated NC. “*I*” stands for intensity. (C) Wide X-ray photoelectron spectroscopy (XPS) spectrum of uncoated NC. “CPS” stands for counts *per* second. (D) Narrow XPS spectrum of the Ce 3d peaks of uncoated NC, with peak deconvolution. (E) Thermogravimetric analysis of uncoated and FBS-coated NC. (F) Dynamic light scattering analysis of NC dispersions in complete cell culture medium after exposure to normal gravity and to simulated microgravity. “HD” stands for hydrodynamic diameter.

Representative electron microscopy images of nanoparticles before and after incubation with FBS are reported in Figure 2A, showing heterodispersed sized nanoparticles with ∼50 nm diameter. In both cases, nanoparticles underwent moderate aggregation after dispersion in an aqueous medium. XRD analysis revealed that: (1) the pattern of uncoated nanoparticles (reported in Figure 2B) is characterized by peaks fully overlapping to the CeO_2_ reference (ICSD 238381), (2) crystallites had a size of 29 nm, and (3) the lattice strain was 0.064%. The value of the lattice strain, which usually correlates to oxygen atom deficiencies, is compatible with the large crystallite size.^51^ XPS analysis confirmed the chemical composition expected in the uncoated nanoparticles: the survey spectrum reported in Figure 2C indeed shows the Ce 3d peaks (relative atomic concentration: 19.5%) in the 875−925 eV range and the O 1s peak (63.9%) at ∼530 eV, together with spurious peaks ascribable to the used substrate (In 3d peaks (0.57%) at ∼444 and 452 eV) and to adventitious carbon (C 1s peak (16.57%) at 284 eV). The narrow spectrum of Ce 3d reported in Figure 2D enabled the quantification of the two oxidation states relevant to the exertion of antioxidant activity *in vitro*: Ce^3+^ (18.8%) and Ce^4+^ (81.2%), as described elsewhere.^52^ A Ce^3+^/Ce^4+^ ratio of 23% is known from the literature to indicate powerful antioxidant activity.^53^ TGA of both uncoated and FBS-coated NC dispersions demonstrated the effectiveness of the nanoparticle coating procedure. As shown in Figure 2E, TGA demonstrated that the native nanoparticles underwent a very modest weight decrease likely ascribable to H_2_O and CO_2_ loss when the temperature increased up to approximately +200 °C, whereas the FBS-coated nanoparticles underwent a more pronounced weight decrease when the temperature increased up to approximately +800 °C, thus denoting relevant adsorption of serum proteins on the nanoparticles with a potential effect on nanoparticle dispersion colloidal stability. Data on the observed weight decrease evidence a difference of ∼17% of weight between uncoated and FBS-coated nanoparticles. DLS analyses, conducted on both uncoated and FBS-coated nanoparticle dispersions over a period of 14 days, demonstrated that the coating procedure determined an increase in the nanoparticle hydrodynamic diameter (HD) and conferred higher colloidal stability to NC in aqueous medium (Figures 2F and S1). Figure 2F reports DLS data acquired on FBS-coated NC dispersed in the cell culture medium, showing comparable values of HD after exposure to both normal gravity and simulated microgravity, suggesting that FBS-coated nanoparticle dispersions were very stable in the observation period, irrespective of the gravity level. ζ-potential analysis was conducted on both uncoated and FBS-coated nanoparticle dispersions in ultrapure water after exposure to different gravity values. As shown in Figure S1, the FBS-coated NC dispersions had comparable, moderately negative values of ζ-potential after exposure to both normal and simulated microgravity, corroborating stability data from HD measurement within the observation period of 14 days.

### Nanoparticle Internalization under Simulated Microgravity

Simulated microgravity studies (with average *g* values ranging from 0.020 to 0.008 within the 6–48 h timeframe) demonstrated that nanoparticles were internalized by cell cultures but, as shown in Figures S2 and S3, NC poorly co-localized with the signal from both internalization markers at each time point and under different gravity levels. Quantitative data reported in Figure S4A,B from confocal microscopy images confirmed modest nanoparticle internalization through pathways mediated by caveolin-1 and clathrin, respectively. It is important to note that a study conducted by Singh and co-workers with nanoceria of different sizes and shapes demonstrated that nanoparticle internalization can be affected by these two geometrical parameters, implying passive internalization when the size is ∼5 nm, and active internalization for higher size nanoparticles.^54^ Due to the heterogeneous size of the NC used in the present work, passive internalization of ∼5 nm nanoparticles may have occurred, whereas larger particles may have undergone other internalization mechanisms (like micropinocytosis). Quantitative data from ICP-OES demonstrate that nanoparticles are internalized by cell cultures under both normal gravity (Ce: 22.5 ± 0.4 ppm) and simulated microgravity (Ce: 1.3 ± 0.3 ppm), although in the latter case at a lower extent than under normal gravity (∼6% of the amount internalized under 1 *g*).

### In-Flight Experiments

**Figure 3:**
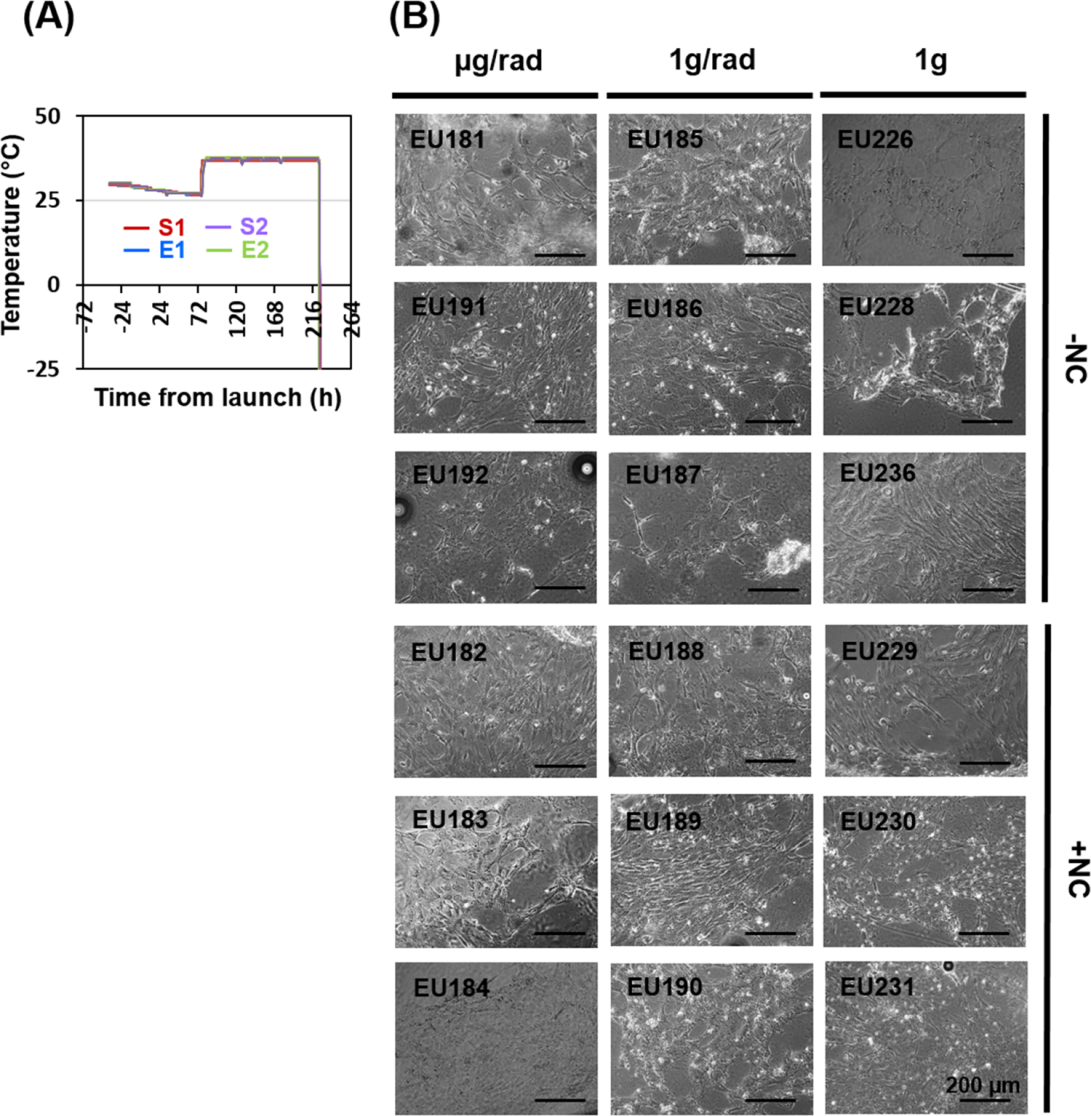
Nanoceria and cell cultures in-flight. (A) Thermal profile of the experiment, where “S” stands for space and “E” for Earth samples. Duplicates are due to the number of data loggers. (B) Representative images, obtained by phase-contrast optical microscopy upon recovery from experiment units (EUs), of cultures exposed to different gravity (g) and radiation (rad) levels, either treated (+NC) or not (−NC) with nanoceria.

As shown in Figure 3A, the cell cultures were exposed to a decreasing temperature between +30 and +26 °C before EU insertion in the Kubik incubator, which occurred at L + 76 h. Viable, adherent, and highly confluent cultures were still obtained at the end of the experimental timeline (Figure 3B). These cultures underwent processing on ground for the following transcriptional analyses.

### RNA Next-Generation Sequencing

The results of analyses performed on nucleic acids after purification are reported in the Supporting Information: Table S1 in particular details the results of spectrophotometric analyses, whereas Figure S5 shows the results of the electropherogram obtained before sequencing. After sequencing, 912 784 532 reads were generated, yielding a total of 213 980 Mb with a mean quality score of 38.61 and a percentage base ≥30 amounting to 93.38. Raw sequence files for each sample are provided as FASTQ files (mRNAs and small RNAs separately).

From the 589 194 183 reads produced altogether by mRNA samples alone, 242 170 173 (∼41%) were successfully mapped to the mouse genome. Of these, 222 402 965 (∼92%) were mapped unambiguously. For mRNA samples, aligned sequences are available as BAM files, and unique gene hit counts are provided as featureCounts output files. Data were deposited in NCBI’s Gene Expression Omnibus database,^55^ and can be accessed through the GEO Series accession number [GSE165565] ([https://www.ncbi.nlm.nih.gov/geo/query/acc.cgi?acc=GSE165565]).

DEG numbers for the nine selected pairwise comparisons among experimental classes (mRNA only) are reported in Table 1. For the most part, a higher number of DEGs was obtained for space-devoted comparisons, with a better statistical significance.

**Table 1:** Summary of Differential Expression Analysis for Protein-Coding Genes^a^

comparison	upregulated genes	downregulated genes	total DEGs
E *vs* F	13	4	17
A *vs* B	23	9	32
C *vs* D	1	0	1
E *vs* A	230	113	343
F *vs* B	383	176	559
E *vs* C	1013	680	1693
F *vs* D	96	77	173
C *vs* A	9	52	61
D *vs* B	4	8	12

a DEG numbers reported here refer to mRNA samples. Experimental classes: A (−NC, μ*g* in space), B (+NC, μ*g* in space), C (−NC, 1 *g* in space), D (+ NC, 1 *g* in space), E (−NC, 1 *g* on ground), and F (+NC, 1 *g* on ground).

**Figure 4:**
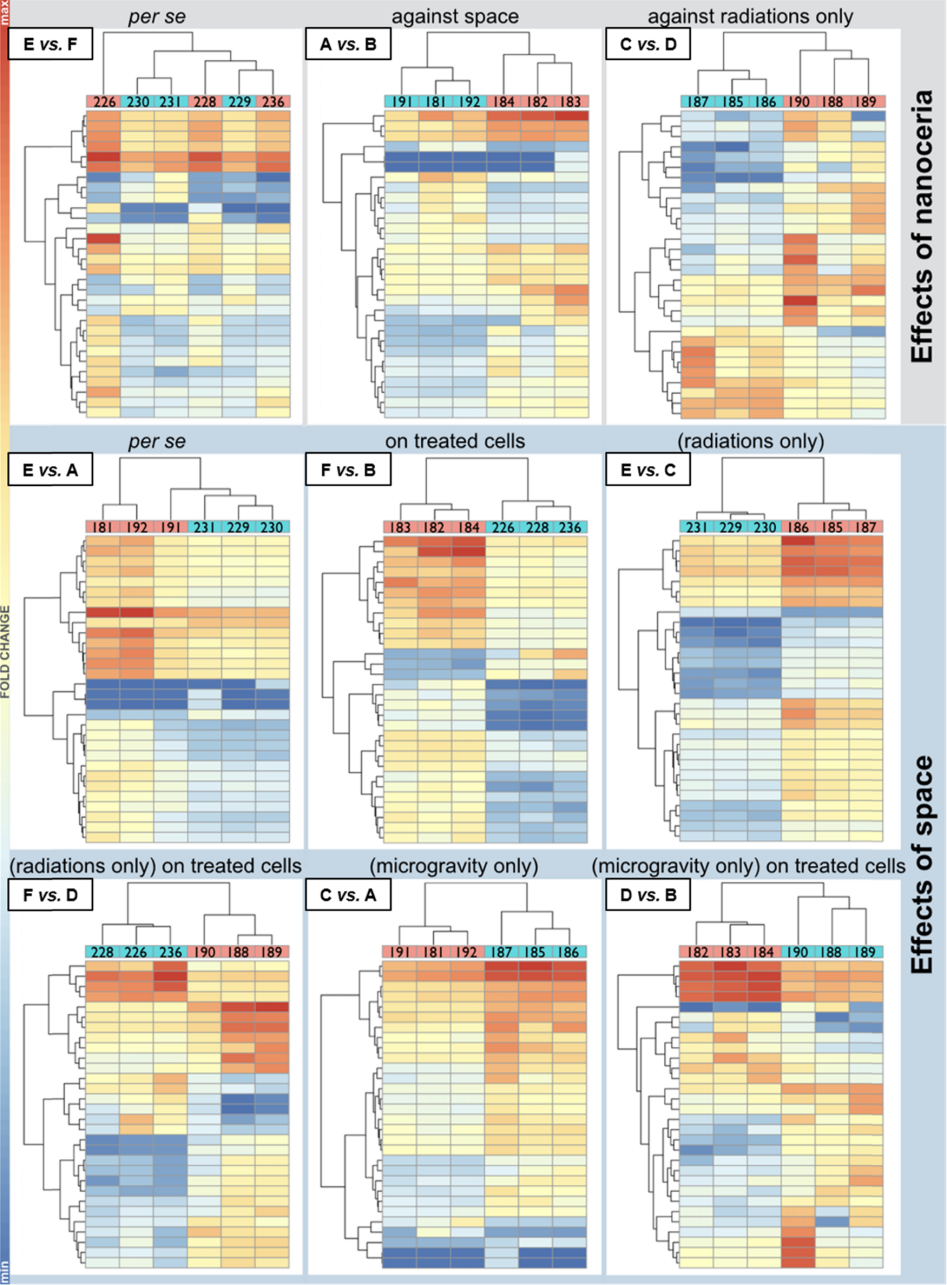
RNA next-generation sequencing heatmaps for the following experimental classes: A (−NC, μ*g* in space), B (+ NC, μ*g* in space), C (−NC, 1 *g* in space), D (+NC, 1 *g* in space), E (−NC, 1 *g* on ground), and F (+NC, 1 *g* on ground). Heatmaps for the nine selected comparisons among experimental classes (mRNAs only, control class reported as the first term of each comparison). For every comparison, a map features (left side of each graph) hierarchical clustering of expression variations for the top 24 genes. Single replicas (EU number is indicated) for the two experimental classes (each in pink or sky blue) are also hierarchically clustered according to the overall expression profile of the 24 genes considered. Expression values for such genes are reported as colored bars. The hotter the color, the higher the fold change (FC; most over-expressed genes within each map are indicated in red, and most under-expressed genes are shown in blue, see the color scale on the left of the panel).

**Figure 5:**
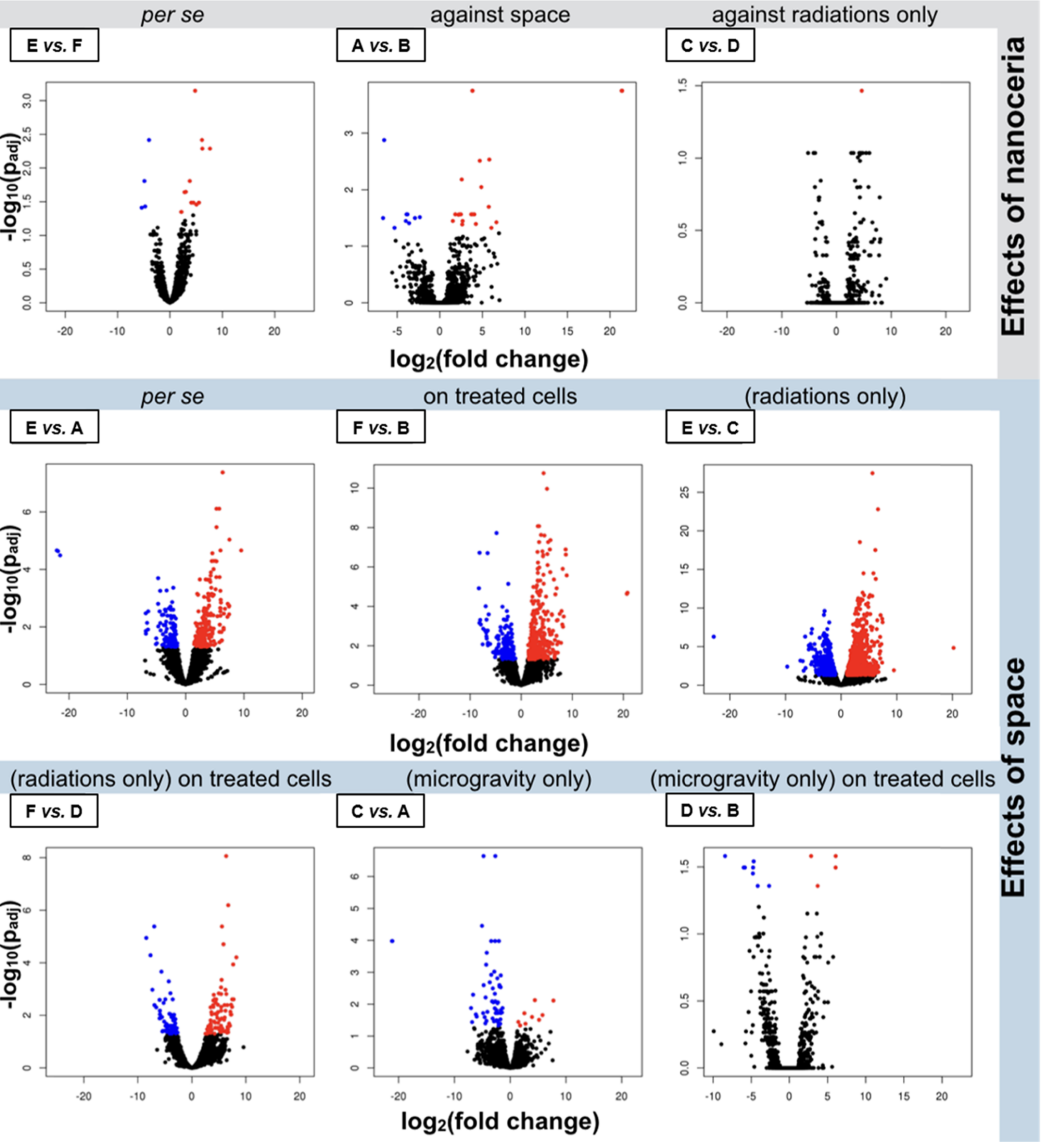
Scatter plots. Volcano plots for the nine selected comparisons among experimental classes (mRNAs only, control class reported as the first term of each comparison). Experimental classes: A (−NC, μg in space), B (+NC, μ*g* in space), C (−NC, 1 *g* in space), D (+NC, 1 *g* in space), E (−NC, 1 *g* on ground), and F (+NC, 1 *g* on ground). Each volcano plot shows significance (*y*-axis, as log_10_ of the adjusted *p*-value, *p*_*adj*_) and overexpression change (*x*-axis, as log_2_ FC, where “FC” stands for fold change) for all genes (each represented by a dot) in a given comparison. Significantly up- or downregulated genes are reported in red (clustering on the right/top corner of the plot) or blue (clustering on the left/top corner of the plot), respectively. The remaining genes are indicated in black.

Heatmaps and volcano plots for the identified DEGs (mRNA only) are respectively shown in Figures 4 and 5.

### Intersections and Gene Ontology

**Figure 6:**
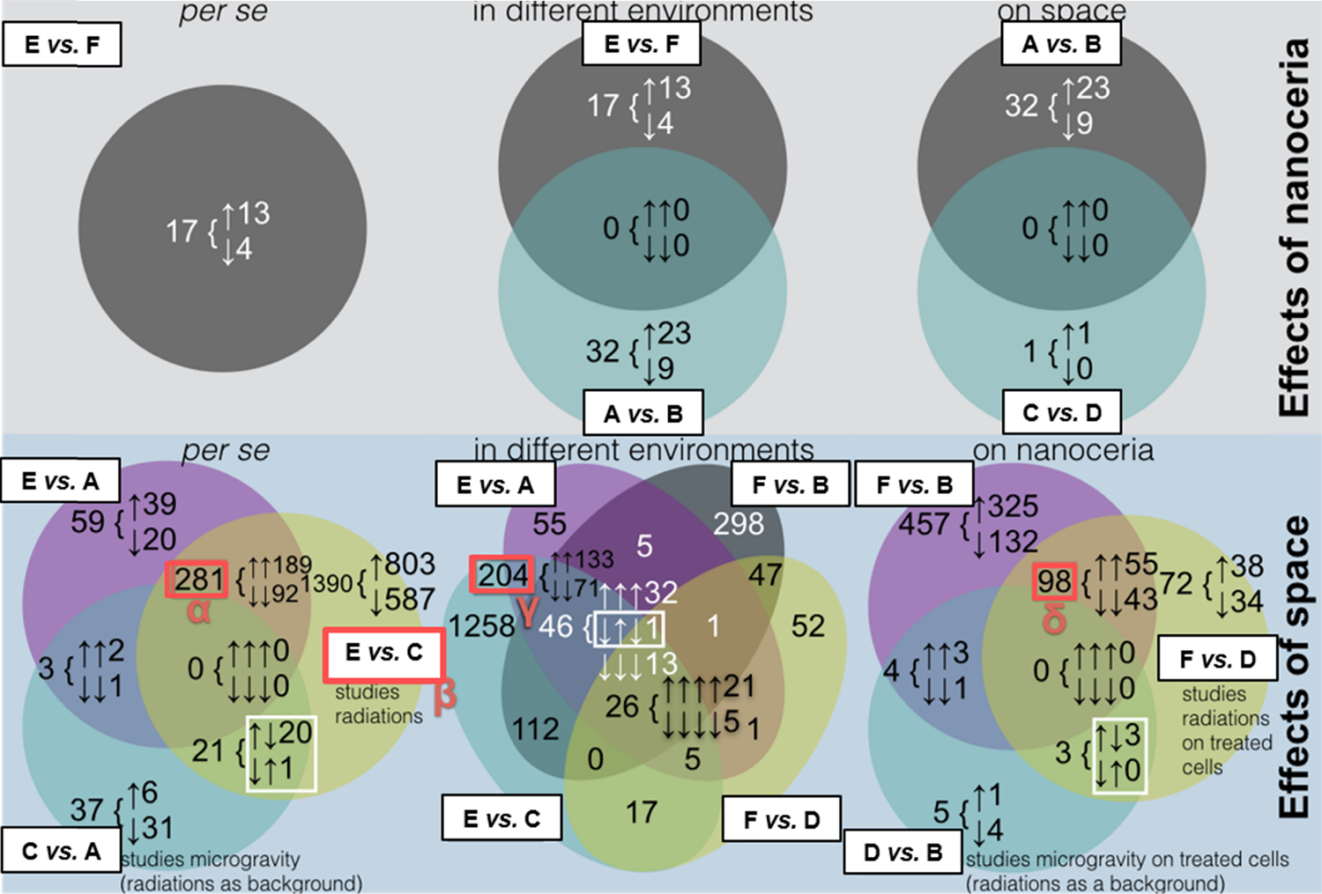
RNA next-generation sequencing Venn diagrams, featuring one to four among the nine selected comparisons among experimental classes (mRNAs only, control class reported as the first term of each comparison). The number of differentially expressed genes (DEGs) residing in each subset is reported. Compatibly with room availability, the composition in terms of up- (↑) or downregulation (↓) is specified after an opening brace: up-only or down-only genes at the intersection between two or more parent sets are deemed coherent. The noncoherent fraction of DEGs at a given intersection, when present and shown, is boxed in white; in that case, to understand in which parent sets a group of DEGs is up- or downregulated, one should assign arrow signs to the relevant parent sets starting from top-left and proceeding in a clockwise order. Intersections for “Effects of nanoceria in different environments” and “Effects of space in different environments” have been simplified, the former not including comparison (C *vs* D) and the latter not considering comparisons (C *vs* A and D *vs* B). Red shadowed boxes, each associated with a Greek letter, highlight particular subsets/parent sets, also depicted by REVIGO interactive graphs.

DEG lists, intersected in different ways to study either NC or space (at times in its microgravity and space radiation components), yielded information about genes recurring as differentially expressed in multiple comparisons and on their transcriptional trends. Figure 6 reports the distribution of DEGs within subsets of the six most relevant Venn diagrams, with details about the internal composition in terms of coherent genes. Overall, when shared between parent sets, the vast majority of DEGs showed a coherent behavior.

**Figure 7:**
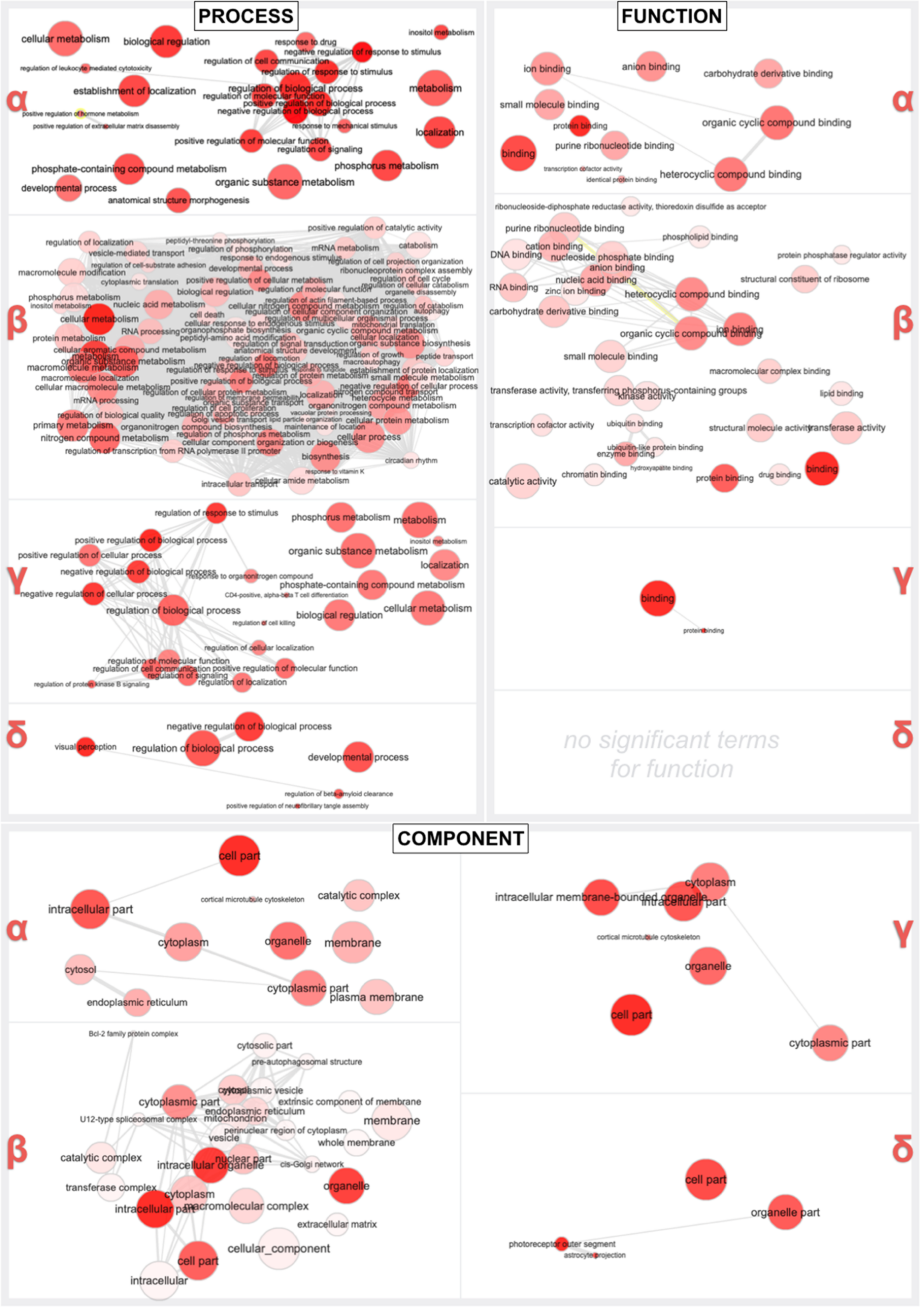
RNA next-generation sequencing gene ontology (GO). REVIGO interactive graphs incorporate enriched (*p*, *q* < 0.05) GO terms for general biological processes (process), specific molecular functions (function), or cell/tissue compartment, niche and thereof (component). Each term is associated with a red circle, which becomes smaller for more specific terms and more saturated for more supported ones. Terms were initially positioned to reflect semantic analogies but were adjusted as needed for graphical reasons. Gray lines connect similar GO terms, with line width reflecting similarity levels.

GO analyses did not conclusively highlight GO terms associated with NC treatment, at least by itself. In turn, space imparts obvious biological effects for both NC-treated and untreated cells, specifically on GO processes related to cell metabolism (e.g., inositol) and localization, on GO functions like binding, and on GO components such as the intracellular domains and organelles (Figure 7).

### Effects of Nanoceria and Space on Muscle Cell Transcriptome

Consistent with past observations from our group,^39^ comparisons that study the effects of NC display a comparatively low number of DEGs. The 17 genes supported as responding to NC on land (E *vs* F) do not show any significant enrichment in GO terms. The corresponding experiments in space (A *vs* B) or in space with gravity (C *vs* D) respectively produce 32 and 1 DEGs, also without significantly associated GO terms. No genes are shared between these sets. Possibly, the moderately lower output of the current investigation for NC could be explained by its more challenging experimental design—namely, deploying actively proliferating cells. In a past work of ours,^39^ downregulation was found as the dominant dynamic among DEGs upon NC treatment, but here around 75% of DEGs are overexpressed when NC is administered (∼77% for E *vs* F, ∼72% for A *vs* B). Space had in turn an evident impact on cell transcriptome, with upregulation being slightly more common and with DEGs exhibiting a strikingly coherent behavior: this is in line with our past results.^39^

Two exceptions are the comparisons that evaluate the impact of microgravity (C *vs* A and D *vs* B): for these, the majority of DEGs are downregulated, and those in common with comparisons studying space radiations reveal a systematically non-coherent trend. Microgravity and space radiations, in other words, seem to cause responses of opposite signs from a single metabolic circuit, albeit no associated GO enrichment was found. When genes are present at the intersection between a comparison for microgravity and others studying space as a whole, coherence is re-established. It should be noticed, however, that all space samples in our study were exposed to ISS space radiations; as a consequence, microgravity is only investigated by subtracting space radiations from space, *i.e.*, with space radiations as part of the experimental environment.

Evaluating the sole effects of space radiations, comparison (E *vs* C) is particularly rich in DEGs. In agreement with a previous report in the literature,^27^ GO graphs for comparison (E *vs* C) generally point to an increased catabolic activity in response to an external factor. Intersected with (E *vs* A), which is the comparison studying space as a whole, the set isolates a group of 281 DEGs, which represent short-listed putative radiation-responsive genes. Their GO plots also hint at metabolic adaptations to exogenous stimuli. Twenty-six genes lie at the intersection between four of the comparisons that investigate space in different ways. As a whole, they do not significantly couple with any GO term, but they still represent potentially interesting candidates for validation studies. The most notable subset of the Venn diagram for the effects of space on NC-treated samples is, perhaps, represented by the 98 genes at the intersection (F *vs* B)/(F *vs* D), another group of possible radiation-sensitive DEGs. An aspect worth mentioning about GO graphs that investigate space radiations is the presence of multiple references to vision. *Rhodopsin* (*Rho*) and its molecular switch *guanine nucleotide-binding protein G(t) subunit α-1* (*Gnat1*) are both present in the 26-gene group of space genes and specifically in the subset of five downregulated genes. One possible explanation is that the exposure to harmful electromagnetic radiations may be somewhat suggestive of light hitting photoexcitable cells.

### ***U**cp2* as a Space-Responsive Gene

The most interesting candidate space genes (eight in total) identified in a past investigation from our group included the *mitochondrial uncoupling protein 2* (*Ucp2*), and this gene was one of the two downregulated coherent factors for space.^39^ In the current dataset, the gene is found among the 281 DEGs responding to space and space radiations in the absence of NC, also by being consistently downregulated. Other studies support *Ucp2* expression as affected by spaceflight or in experimental procedures modeling altered gravity.^39^

Ucp2 is abundant in skeletal muscle. It was once thought to be mainly active in non-shivering thermogenesis. However, studies on mutant mice conclusively show that the protein is primarily a sensor and detoxifier of mitochondrial ROS. It is highly represented also in the spleen and leukocytes, and it is necessary for the maintenance of normal immune and inflammatory responses. In particular, it plays a role in macrophage-mediated immunity.^56^

A secondary physiological function of Ucp2 is erythropoiesis: a dysfunctional Ucp2 leads to anemia.^57^ The protein also protects from ischemia, atherosclerosis, myocardial infarction, and several degenerative disorders.^58^ Muscle waste, anemia, and immune pathologies (including macrophage-dependent ones) are conditions often observed during prolonged spaceflight.^26,59^ Intriguingly, mouse models show that a molecular mechanism linking Ucp2 to anemia is the MAPK/ERK pathway, with the lack of Ucp2 causing a decrease in the active form of ERK, a ROS-triggered regulator of proliferation.^57,60^ The pathway has recently been found to be altered in space or space analogues, directly generating at least some of the macrophage pathologies typical of spaceflight.^59^ ERK exerts in reverse a negative feedback on Ucp2, as shown in cell cultures.^60^*Ucp2* is known to respond to both chronic and short-term OS, controlling the oxygen metabolism of the cell and, therefore, limiting the amount of radicals produced by oxidative phosphorylation; it is thus a promising therapeutic target for ROS-elicited disease.^58,61^

## Conclusions

The transcriptional evidence collected by the application of an experimental protocol involving antioxidant nanoparticle administration to muscle cell cultures during spaceflight (meaning exposure to different gravity levels and to cosmic radiations) demonstrated the prevalence of space effects over those of antioxidants. Under our experimental conditions, space radiations in particular emerged as an impacting force on muscle cell transcriptome. Synergies between microgravity and space radiations have been investigated previously, with somewhat contradictory outcomes.^27^ Our results suggest that the two stressors act through partially overlapping molecular components with an evocation of opposite responses, and that there might be an analogy between photoreception and response to space radiations deserving further studies also at molecular level. Along with previous evidence from our group, the current study corroborates the concept of OS being a relevant dynamic behind space pathology. Our data indeed reinforce the role of *Ucp2* as a key mediator of cell response to space, either on its own or in conjunction with factors of the MAPK/ERK signaling pathway. Future studies will therefore aim at optimizing antioxidant administration in space and at fully elucidating the role of *Ucp2* as a promising molecular target for the treatment of spaceflight-induced tissue waste.

## Supporting Information Available

Colloidal stability results of both uncoated and FBS-coated NC dispersions in different aqueous media upon exposure to both normal gravity and simulated microgravity (Figure S1); representative confocal images for NC internalization studies after cell culture exposure to both normal gravity and simulated microgravity (Figure S2, caveolin-1 immunostaining) and (Figure S3, clathrin immunostaining); results from internalization studies by immunostaining and confocal microscopy (Figure S4); a bioanalyzer (Agilent) gel-like picture of RNA preparations obtained from space and ground samples before sequencing (Figure S5); results of spectrophotometric analysis on the same samples (Table S1) (PDF)

## Abbreviations Used

DEG: differentially expressed gene
DLS: dynamic light scattering
EC: experiment container
ESA: European Space Agency
EU: experiment unit
FBS: fetal bovine serum
FC: fold change
GO: gene ontology
HD: hydrodynamic diameter
ICP-OES: inductively coupled plasma-optical emission spectroscopy
ISS: International Space Station
KISS: Kubik Interface Simulation Station
L: launch time
MELFI: Minus Eighty-Degree Laboratory Freezer for ISS
NASA: National Aeronautics and Space Administration
NC: nanoceria
RNA-seq: RNA next-generation sequencing
OS: oxidative stress
PCR: polymerase chain reaction
ROS: reactive oxygen species
s-μg: simulated microgravity
TEM: transmission electron microscopy
TGA: thermogravimetric analysis
XPS: X-ray photoelectron spectroscopy
XRD: X-ray diffraction
