# Supplementary material for: Cerium Oxide Nanoparticle Administration to Skeletal Muscle Cells under Different Gravity and Radiation Conditions
Source: ACS Appl Mater Interfaces. 2021 Aug 19;13(34):40200–13. doi: 10.1021/acsami.1c14176 (PMC8414486; doi:10.1021/acsami.1c14176)
Supplement: Supplementary file 1 — am1c14176_si_001.pdf [file am1c14176_si_001.pdf]

SUPPORTING INFORMATION FOR

Cerium oxide nanoparticle administration to skeletal muscle cells under different gravity and radiation conditions

Giada Graziana Genchi[a,*,‡], *Andrea Degl'Innocenti*[a,*,‡], *Chiara Martinelli*[a,*,‡], *Matteo Battaglini*[a], *Daniele De Pasquale*[a,b], *Mirko Prato*[c], *Sergio Marras*[c], *Giammarino Pugliese*[d], *Filippo Drago*[d], *Alessandro Mariani*[e], *Michele Balsamo*[e], *Valfredo Zolesi*[e], *Gianni Ciofani*[a,*]

[a]Istituto Italiano di Tecnologia, Smart Bio-Interfaces, Viale Rinaldo Piaggio 34, 56025 Pontedera (Pisa), Italy

[b]Scuola Superiore Sant'Anna, The BioRobotics Institute, Viale Rinaldo Piaggio 34, 56025 Pontedera (Pisa), Italy

[c]Istituto Italiano di Tecnologia, Materials Characterization, Via Morego 20, 16163 Genova, Italy

[d]Istituto Italiano di Tecnologia, Nanochemistry, Via Morego 20, 16163 Genova, Italy

[e]Kayser Italia S.r.l., Via di Popogna 501, 57128 Livorno, Italy

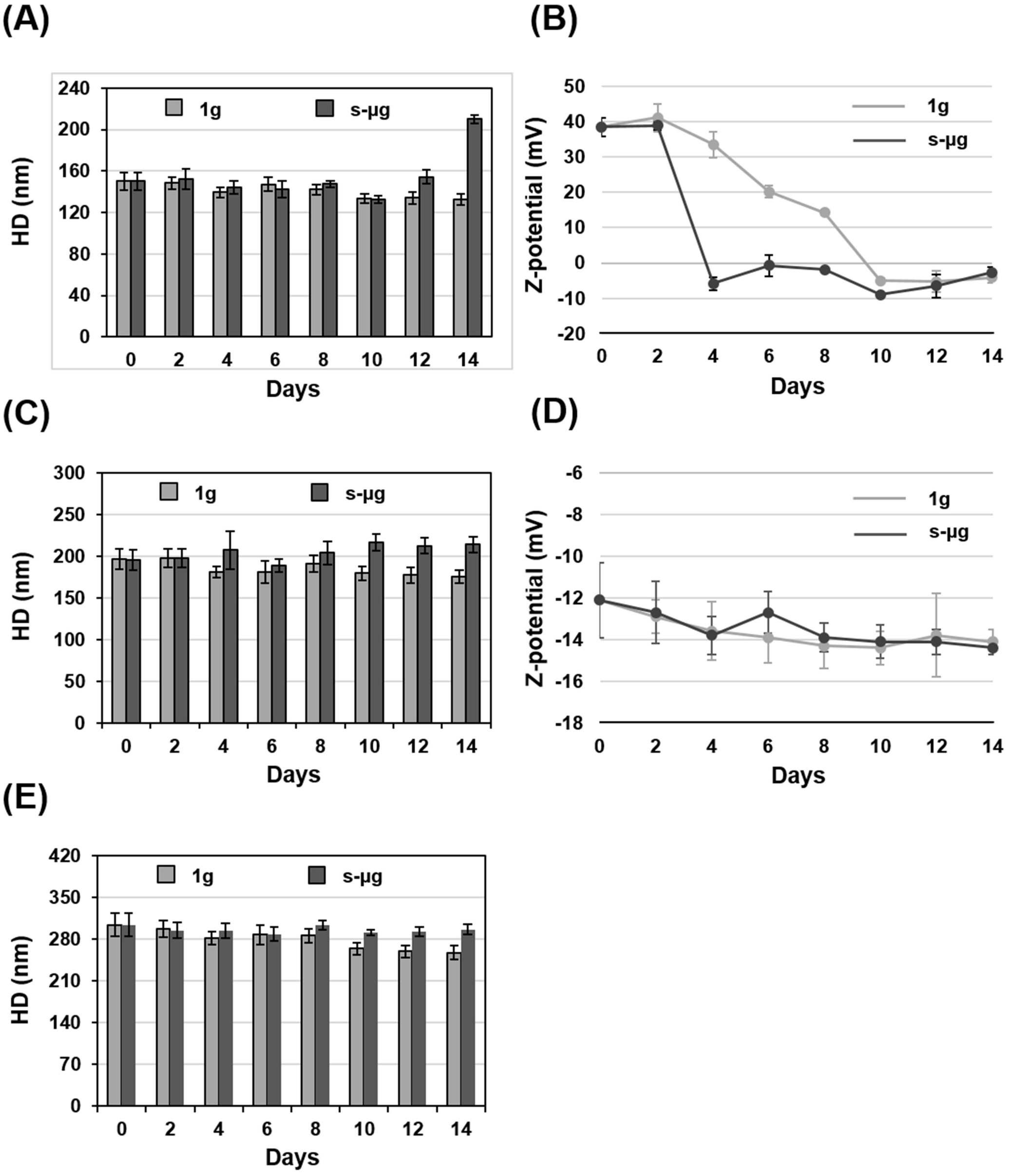

Figure S1. Characterization of cerium oxide nanoparticle (NC) dispersions. (A) Dynamic light scattering (DLS) analysis of NC dispersions in ultrapure water after exposure to normal gravity and to simulated microgravity. (B) Z-potential measurements of NC dispersions in ultrapure water after exposure to normal gravity and to simulated microgravity. (C) DLS analysis of FBS-coated NC dispersions in ultrapure water after exposure to normal gravity and to simulated microgravity. (D) Z-potential measurements of fetal bovine serum (FBS)-coated NC dispersions in ultrapure water after exposure to normal gravity and to simulated microgravity. (E) DLS analysis of FBS-coated NC dispersions in 10% FBS solution in ultrapure water after exposure to normal gravity and to simulated microgravity. "HD" stands for hydrodynamic diameter, and "s-µg" stands for simulated microgravity.

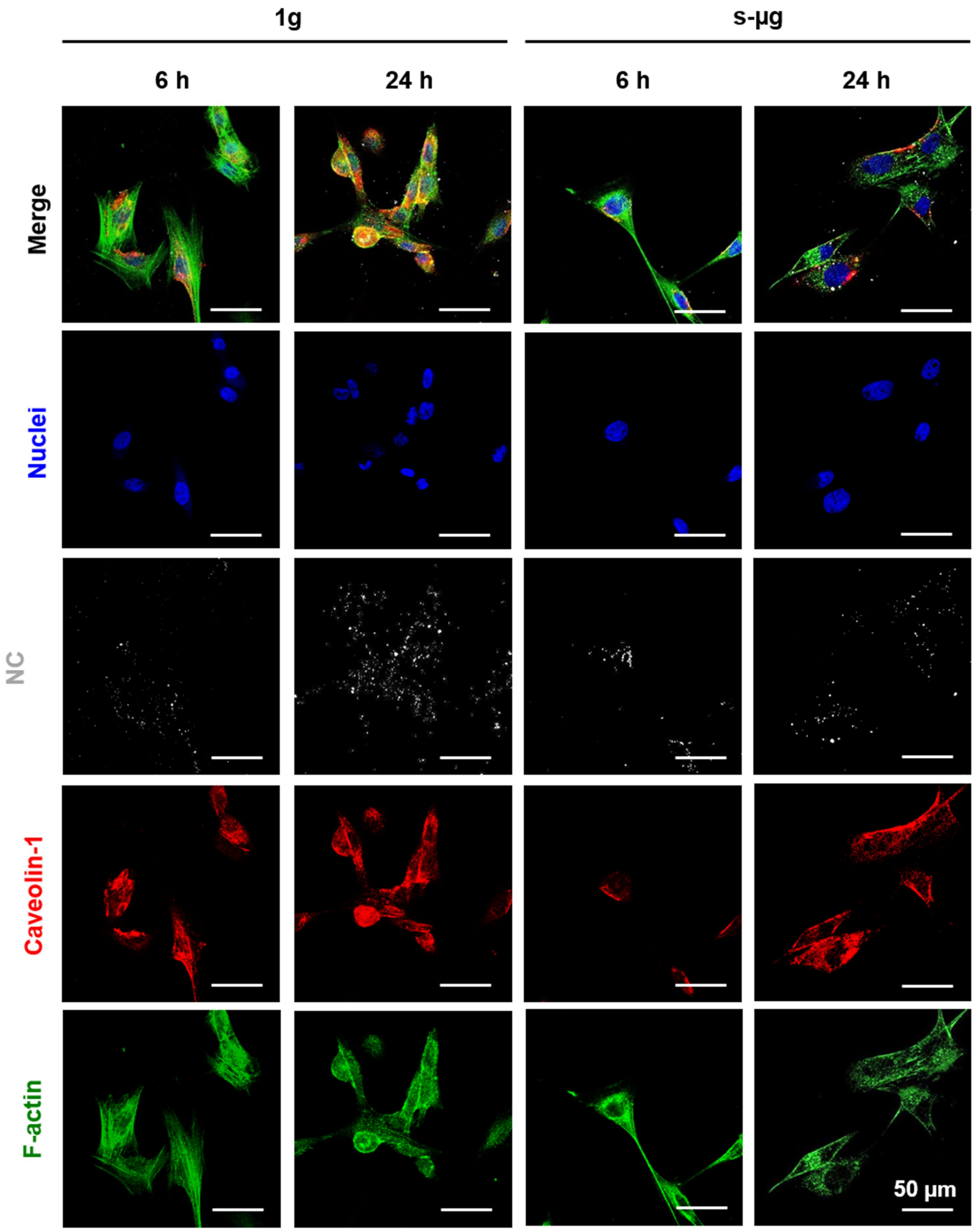

Figure S2. Internalization study through immunofluorescence staining of caveolin-1 and confocal microscopy imaging. Nanoparticle signal originates from light scattering. "NC" stands for nanoceria; "s-μg" stands for simulated microgravity.

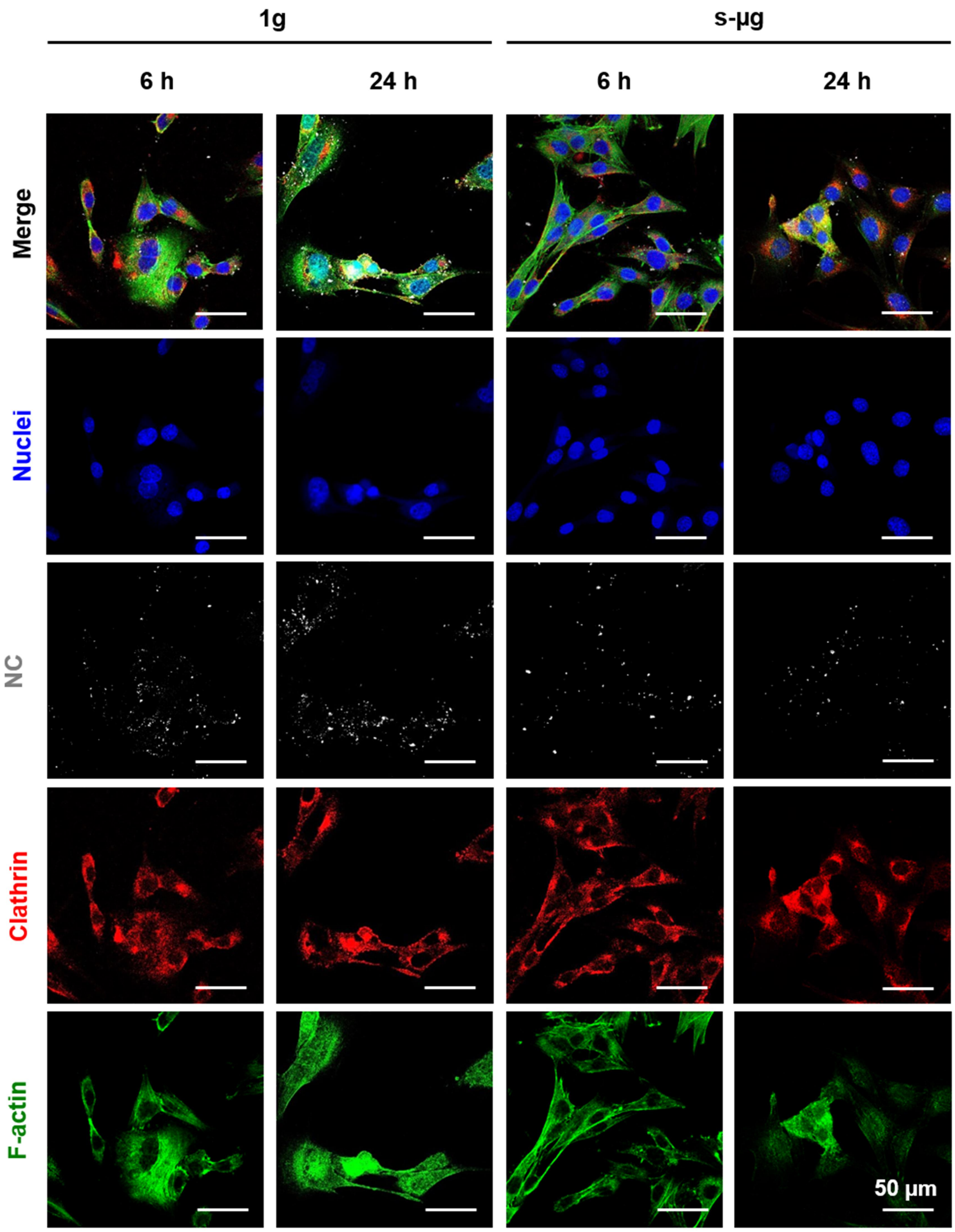

Figure S3. Internalization study through immunofluorescence staining of clathrin and confocal microscopy imaging. Nanoparticle signal originates from light scattering. "NC" stands for nanoceria; "s-μg" stands for simulated microgravity.

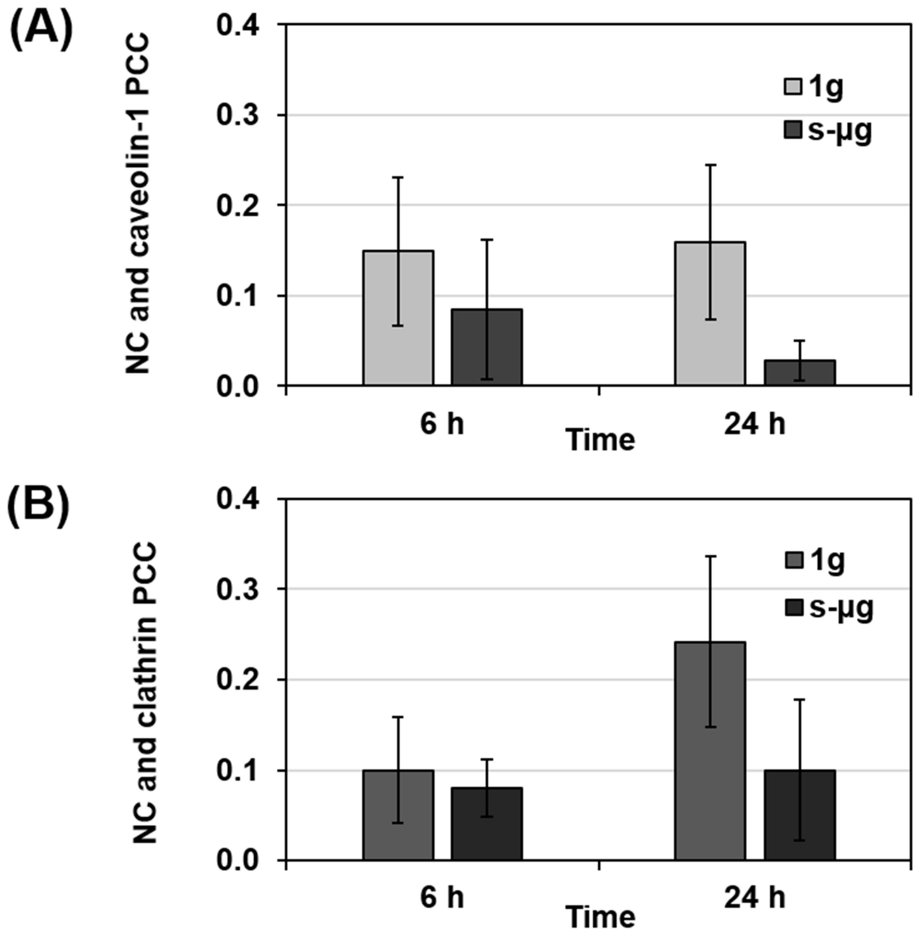

Figure S4: Internalization study. (A) Histograms depicting Pearson's correlation coefficient (PCC) measuring co-localization of signal from nanoceria (NC) and caveolin-1 in cell cultures, exposed to either normal gravity or simulated microgravity for 6 h and 24 h. (B) Histograms depicting PCC measuring co-localization of signal from NC and clathrin in cell cultures exposed either to normal gravity and to simulated microgravity for 6 h and 24 h. "s-µg" stands for simulated microgravity; "+NC" stands for nanoceria-treated; "-NC" stands for non-nanoceria-treated. Data are represented as average ± standard deviation (n= 3).

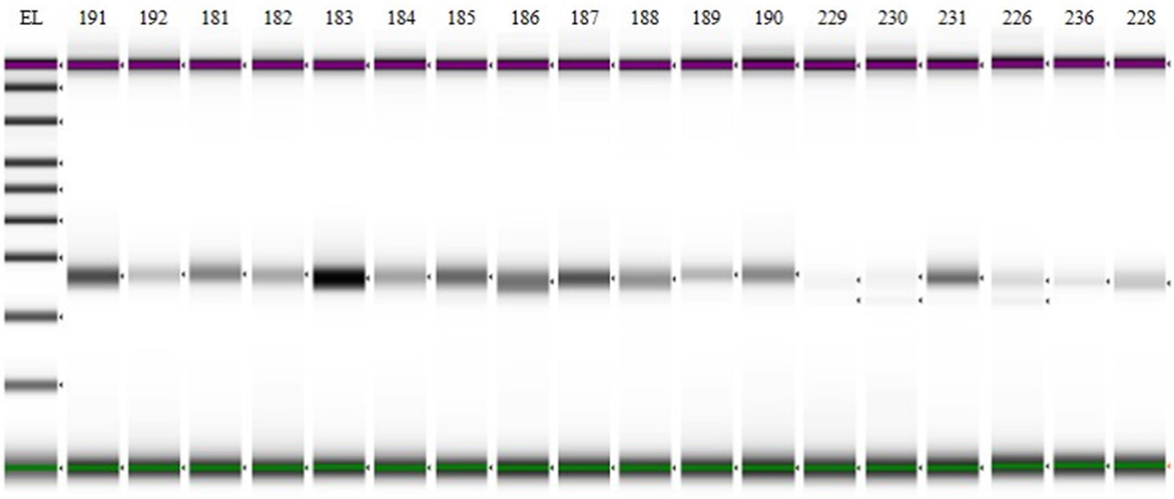

Figure S5. RNA quality assessment obtained by Bioanalyzer (Agilent). “EL” stands for electronic ladder. Numbers indicate each experiment unit.

Table S1. Photometric analysis of total RNA after purification.

Place	EU	Treatment	Gravity regimen	RNA (ng/µl)	A260	A280	A260/ A280	A260/ A230
ISS	191	-NC1	µg	23.6	0.591	0.299	1.98	0.35
ISS	192	-NC2	µg	12.2	0.304	0.152	2.00	0.15
ISS	181	-NC3	µg	9.6	0.240	0.125	1.92	0.11
ISS	182	+NC1	µg	13.6	0.341	0.187	1.82	0.73
ISS	183	+NC2	µg	9.3	0.231	0.123	1.88	0.41
ISS	184	+NC3	µg	5.9	0.148	0.085	1.74	0.12
ISS	185	-NC1	1g	21.8	0.544	0.283	1.93	0.48
ISS	186	-NC2	1g	20.5	0.513	0.248	2.07	0.18
ISS	187	-NC3	1g	9.9	0.247	0.131	1.89	0.27
ISS	188	+NC1	1g	10.4	0.260	0.134	1.95	0.20
ISS	189	+NC2	1g	14.1	0.353	0.154	2.29	0.17
ISS	190	+NC3	1g	17.4	0.435	0.202	2.15	0.17
IIT	229	-NC1	1g	7.6	0.190	0.089	2.14	0.09
IIT	230	-NC2	1g	5.4	0.135	0.077	1.76	0.19
IIT	231	-NC3	1g	9.0	0.225	0.144	1.56	0.35
IIT	226	+NC1	1g	4.8	0.119	0.083	1.44	0.25
IIT	236	+NC2	1g	5.2	0.130	0.084	1.55	0.05
IIT	228	+NC3	1g	5.1	0.127	0.083	1.53	0.37

"A" stands for absorbance at either 260 (A260) or 280 (A280) nm; "EU" stands for experiment unit. "IIT" stands for Istituto Italiano di Tecnologia; "ISS" stands for International Space Station. The string defining sample treatment starts with a notation indicating whether a sample has been treated with nanoceria (+NC) or not (-NC), followed by the replicate number. For instance, "+NC1" means nanoceria-treated replicate 1.
